# Beta-alanine supplementation improves time to exhaustion, but not aerobic capacity, in competitive middle- and long-distance runners

**DOI:** 10.1080/15502783.2025.2521336

**Published:** 2025-06-17

**Authors:** David Marko, Ronald L. Snarr, Petr Bahenský, Václav Bunc, Miroslav Krajcigr, Tomáš Malý

**Affiliations:** a Charles University, Sports Motor Skills Laboratory, Faculty of Sports, Physical Training and Education, Prague, Czech Republic; b University of South Bohemia in České Budějovice, Department of Sports Studies, Faculty of Education, České Budějovice, Czech Republic; c Texas A&M University – Corpus Christi, Department of Kinesiology, Corpus Christi, TX, USA; d Charles University Prague, Department of Physical Education, Faculty of Education, Prague, Czech Republic

**Keywords:** Dietary supplements, running performance, VO_2peak_, VO_2max_, aerobic performance

## Abstract

**Background:**

Beta-alanine (βA) is a non-essential amino acid purportedly used to enhance aerobic exercise performance. While previous research indicates the benefits of βA on time to exhaustion (TTE) and aerobic capacity (VO_2peak_) in adults, evidence is lacking in adolescent athletes. Thus, the purpose of this study was to determine the effects of 4 weeks of βA supplementation on aerobic performance in adolescent runners.

**Methods:**

Twenty-seven middle- and long-distance runners (aged 17.36 ± 2.17 years) were randomly divided into a βA or placebo (PL) group (maltodextrin). Subjects performed maximal graded exercise tests (GXT) and submaximal trials (SMT; 80% of VO_2peak_ for 1500 m) on a treadmill before and after 14 and 28 days of supplementation or PL. Respiratory (V_E_) metabolic (VO_2_, RER, lactate [La]), and cardiovascular (HR) variables were measured during the GXT and SMT, along with the first (VT1) and second ventilatory threshold (VT2) and TTE monitored during the GXT only. Within- and between-group differences were assessed using a repeated-measures mixed-model analysis of variance.

**Results:**

Findings indicated that despite a trivial increase in VO_2peak_ over 4 weeks, the βA group increased TTE by 6.5% compared to 1.4% in the PL group (*d* = 0.46). Additionally, small effects in HR_max_, V_E_, [La], and TTE were observed between groups favoring βA. Regarding the SMT, both average HR and RER decreased by 4% in the βA group, with no changes for the PL.

**Conclusions:**

Despite no evidence to suggest increases in VO_2peak_, practitioners should note that improvements in TTE may be observed after 28 days of βA supplementation in adolescent runners.

## 1. Introduction

One of the largest physiological contributors to fatigue during exercise is the accumulation of hydrogen ions (H^+^) within the skeletal muscle tissue and bloodstream [1–4]. Due to the accumulation of H^+^, there is a subsequent reduction in pH, which has been shown to disrupt the resynthesis of phosphocreatine [1], inhibit glycolysis [3], and impair the transfer of ions (e.g. lactate) across cellular membranes [4]. These disruptions collectively contribute to an early onset of fatigue and impair exercise performance. Therefore, to combat these detrimental effects, attenuate reductions in pH, stave off fatigue, and ultimately improve performance, athletes have resorted to consuming dietary supplements designed to buffer H^+^ (e.g. β-alanine [βA]) [5–7]. While βA has many functions (e.g. antioxidant, neurotransmitter) its primary role during exercise is to increase the synthesis of carnosine, a dipeptide responsible for buffering H^+^ [2,5,6,8]. With a consistent consumption of supplemental βA (typically ranging from 3.2 to 6.4 g·day^– 1^) for 4 weeks and longer, research has demonstrated elevations of carnosine levels by ~20–80% and improved H^+^ buffering capacity of 6–7% on average [9–11]. Thus, βA supplementation has the potential to improve exercise performance, particularly for events producing higher levels of H^+^ (e.g. high-intensity exercise).

While increasing the buffering capacity for H^+^ ions would not solely alleviate fatigue during exercise performance, multiple studies have demonstrated beneficial effects when consuming βA including delayed onset of neuromuscular fatigue, improved time to exhaustion (TTE), and increased total work completed [6,11–14]. For instance, after 4 weeks of βA supplementation (with doses progressively increasing from 4.0 to 6.4 g/day), recreationally trained college-aged males increased total work completed during high-intensity cycling trials (at 110% of maximum power) by an average of 13% (i.e. 7.3 ± 1.3 kJ) compared to 2.3% (i.e. 1.1 ± 1.1 kJ) in the control [11]. Similarly, physically active males undergoing a similar protocol (i.e. high-intensity cycling) improved TTE by 12.1% after 4 weeks of βA supplementation (6.4 g/day), compared to a 1.6% increase for the placebo group [15]. Four weeks of βA supplementation (administered as 6.4 g/day for the first 6 days followed by 3.2 g/day for the remaining 22 days) also delayed the onset of neuromuscular fatigue in untrained males during incremental cycle ergometry by 14.5% [13]. Additionally, 4 weeks of βA supplementation (beginning with 3.2 g·day^–1^ in week one and increasing to 6.4 g·day^–1^ from week 2 onward) improved TTE in continuous, incremental cycle ergometry by 2.6% in adult females [14].

Although previous literature has demonstrated favorable increases in TTE when supplementing with βA, findings regarding improvements in aerobic capacity (VO_2peak_) and other performance variables (e.g. sprinting) are inconsistent [6,12,16]. For instance, after completing 6 weeks of βA supplementation (administered as 6 g/day for the first 21 days followed by 3 g/day for the remaining 21 days) and high-intensity interval training on a cycle ergometer, recreationally active males exhibited similar increases in VO_2peak_ (i.e. 11.9%) compared to a placebo group (i.e. 12.6%) [12]. In addition, while no statistical change in VO₂_peak_ was observed following 28 days of β-alanine supplementation at 6 g/day in recreationally active individuals, individuals consuming βA exhibited large practical improvements in physical working capacity at heart rate threshold (+24.2 W) compared to placebo (+11.2 W), indicating enhanced submaximal performance [17]. Not limited to VO_2peak_, elite male sprinters observed a ~0.7 s decrease in 400-m running performance after 5 weeks of βA supplementation at 4.8 g/day, despite a 47% increase in the skeletal muscle carnosine [6]. The 0.7 s decrease in sprint time was consistent with the results reported from the placebo group, indicating that βA supplementation had no effect on performance.

Although βA supplementation has traditionally been associated with improvements in high-intensity and anaerobic performance, its role in endurance exercise is less well-defined. Endurance-based events frequently contain intermittent bouts of higher intensity activities (e.g. surges, uphill sections, final sprints), during which H^+^ accumulation may impair performance. Moreover, improved muscle buffering capacity may contribute to maintaining submaximal performance for longer durations, delaying the onset of fatigue during prolonged efforts. Thus, investigating the effects of β-alanine in an endurance-based context – especially among competitive runners – represents a relevant and understudied area of inquiry. Yet, despite the abundance of evidence demonstrating alterations, or inconsistent alterations, in performance for adult athletes, to the authors knowledge, there are no studies addressing the use of βA in adolescent competitive runners. Accordingly, the purpose of the current study was to investigate 4 weeks of βA supplementation on submaximal and maximal endurance performance in elite adolescent runners. Based on previous findings, and the ability of βA to increase carnosine levels and buffer H^+^ ions during aerobic-based exercise, it was hypothesized that subjects would experience increases in time-to-exhaustion with no accompanying rise in aerobic capacity.

## 2. Methods

### 2.1. Experimental approach to the problem

The current study used a double-blind, placebo-controlled pre-posttest design that examined the effect of 4 weeks of βA supplementation on the metabolic, cardiovascular, and respiratory responses to submaximal and maximal exercise in elite adolescent runners (aged 15–19 years). Twenty-seven middle- and long-distance runners (i.e. 800 m, 1500 m, 3000 m) visited the Laboratory of Load Diagnostics to complete three submaximal and three maximal treadmill testing sessions to exhaustion, over a 4-week period, while ingesting either βA or a placebo (PL) daily. During the submaximal tests, each of the following were collected: average and final HR, average (VO_2avg_) and final oxygen uptake (VO_2final_), post-exercise blood lactate concentration measured 3 min after test cessation ([La]), respiratory exchange ratio (RER), rate of perceived exertion (RPE), body weight, and height. During each maximal treadmill test, the following variables were measured: maximal heart rate (HR_max_), VO_2peak_, velocity (*v*), 3-min posttest [La], TTE, minute ventilation (V_E_), ventilatory threshold 1 (VT1), ventilatory threshold 2 (VT2), RER, RPE, body weight, and height. To maintain consistency, both groups (i.e. βA and PL) completed the same training program over the four-week intervention and were also asked to remain on their current dietary regimen to reduce variance in βA intake from changes in nutritional habits.

### 2.2. Subjects

Twenty-seven adolescent middle- and long-distance runners participated in this study (males: *n* = 13; females: *n* = 14). To be included, subjects had to be training for at least 3 years as a competitive runner, have not used dietary supplements within the last 3 months, experienced no injuries within the last 6 months, and between the ages of 15–19 years. Individuals were excluded if they had any metabolic, cardiovascular, or neurological disorders, as well as any conditions that would be exacerbated by the research protocol. Prior to any testing, written consent, and parental assent (when applicable), was obtained for each subject. The current research was performed with compliance of the ethical standards of the Institutional Research Committee, Helsinki Declaration, and the University of South Bohemia (Ref. No.: 026/2023).

### 2.3. Protocol

**Figure 1.**
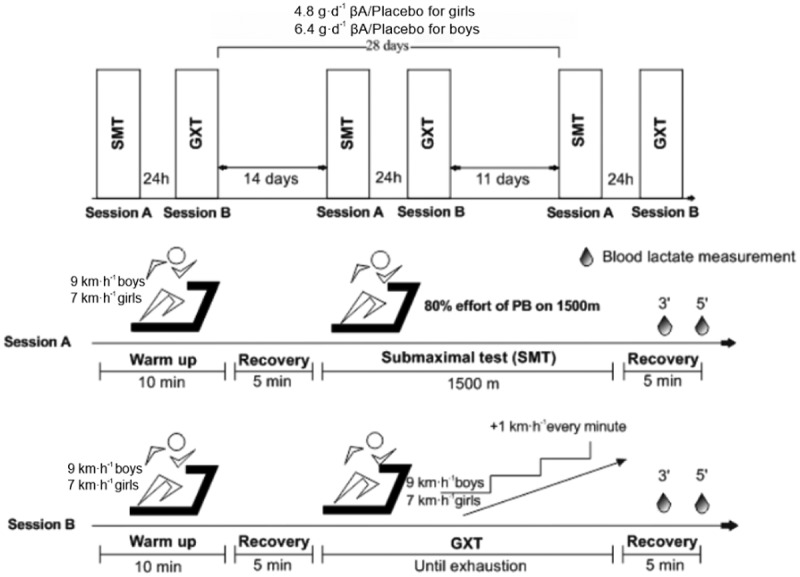
Study design to determine the effects of β-alanine supplementation (versus placebo) on submaximal and maximal aerobic endurance performance in elite adolescent runners.

For this study, subjects were asked to visit the laboratory six times over a four-week period to complete three submaximal time trials (SMT) and three maximal graded exercise tests (GXT) (Figure 1). Over the four-week period, subjects also consumed either βA (4.8 g·d^–1^ for females and 6.4 g·d^–1^ for males) or PL (i.e. 4.8 g·d^–1^ of maltodextrin for females and 6.4 g·d^–1^ for males), in pill form, on a daily basis. All exercise testing sessions were performed on a treadmill (Lode Valiant 2 Sport, Lode B.V., Groningen, The Netherlands) with all bouts occurring at the same time of day for each participant to maintain consistency. Additionally, subjects were familiarized with all testing procedures prior to data collection to minimize the learning effect. During each SMT and GXT, respiratory (i.e. V_E_), metabolic (i.e. VO_2_, RER, [La]), and cardiovascular (i.e. HR) variables were measured using breath-by-breath metabolic analysis (Metalyzer B3, Cortex, Leipzig, Germany) and heart rate monitoring, via chest strap (Polar H7, Polar Electro Oy, Kemple, Finland). However, during the GXT, additional variables (i.e. VT1, VT2, VO_2peak_, TTE) were monitored to further determine the effects of βA supplementation on aerobic performance. For this investigation, VT1 was calculated as the increase in both the ratio between minute ventilation and oxygen consumption (V_E_/VO_2_) and end-expiratory oxygen tension (P_ET_O_2_) with no associated increase in the ratio of minute ventilation and volume of expired carbon dioxide (V_E_/VCO_2_) [18]. VT2 was estimated via the simultaneous increase in both V_E_/VO_2_ and V_E_/VCO_2_ with a decrease in end-expiratory partial pressure of carbon dioxide (P_ET_CO_2_) [19].

βA: β-Alanine; GXT: graded exercise test; SMT: Submaximal test; Placebo: Maltodextrin (6.4 g/day for boys, 4.8 g/day for girls); PB: Personal best on 1500 m time trial.

To estimate metabolic stress, [La] was collected three- and five-minutes post submaximal and maximal testing using a Lactate Scout 4 analyzer (SensLab GmbH, Leipzig, Germany). La was collected 3-min post testing as peak [La] values are typically at their highest concentration during this period post-exercise [20]. La samples were collected from the left index finger, which was cleaned with an alcohol pad and dried with gauze prior to sampling.

### 2.4. Submaximal testing (SMT)

Subjects performed three SMTs over the course of a four-week intervention (i.e. baseline, week 2, and week 4). Each SMT consisted of a 1500-m treadmill run set at an intensity equal to 80% of their individual VO_2peak._ The VO_2peak_ of all runners was determined using a GXT, taken on the first visit to the laboratory. The SMT was preceded by a standardized 10-min warm-up (i.e. 7 km·h^–1^ for females and 9 km·h^–1^ for males) followed by a five-minute break. Following the break, subjects completed the 1500-m run while wearing the metabolic analyzer mask and heart rate chest strap. Once completed, the individuals had a 5-min active recovery period, at 3 km.h^–1^, with [La] collected at 3-min post during the recovery.

### 2.5. Graded exercise testing (GXT)

Subjects completed three GXTs over the four-week intervention (i.e. baseline, week 2, and week 4). Each GXT began with a standardized 10-min warm-up of 9 km·h^–1^ for males and 7 km·h^–1^ for females, followed by a 5-min break. After the break, the treadmill speed was set to 7 km·h^–1^ for females and 9 km·h^–1^ for males with an increase of 1 km·h^–1^ every minute, and a constant grade of 5%, until volitional exhaustion. The initial speed was determined based on previous studies with the intent of eliciting fatigue within 8–12 min. Once the subjects reached volitional fatigue, a 5-min active recovery period of treadmill walking, at a speed of 3 km.h^–1^, was completed with [La] measured at 3-min posttest. GXT termination criteria for attainment of VO_2peak_ included satisfying three of the following: 1) plateau in VO_2_ (<2.1 ml·kg^–1^∙min^–1^ increase) despite an increase in workrate during the final two stages of the test; 2) respiratory exchange ratio >1.1; 3) rating of perceived exertion >17 on a 6–20 scale; 4) achievement of 90% of age predicted HR_max_; and 5) [La] of ≥8 mm/L [16].

### 2.6. Supplementation

Subjects were randomly assigned to either a PL or βA group in a double-blind manner using a computer-generated randomizer [21]. The supplement group was provided with commercially available βA (GymBeam, Berlin-Gartenfeld, Germany) (6.4 g·d^–1^ of βA for males, 4.8 g·d^–1^ βA for females), while the PL group was provided with either 6.4 g·d^–1^ of maltodextrin for males or 4.8 g·d^–1^ of maltodextrin for females. To our knowledge, there have only been four studies published involving βA supplementation and adolescents [22–25]. All four studies were carried out on adolescent males and subjects consumed 6.4 g·d^−1^ of βA or PL, approximately 90 mg·kg^−1^. Therefore, the dosage for adolescent females was set to 4.8 g·d^−1^ of βA or PL, approximately 90 mg·kg^−1^. All runners were instructed to consume the βA or PL three times per day with meals [16]. For compliance with the dosage protocols, subjects were messaged three times per day, using the Whatsapp™ application, to confirm consumption. Individuals were excluded from the study after not taking the supplement for 1 day (i.e. three doses).

### 2.7. Statistical analysis

All data were analyzed using SPSS version 28 (IBM Corp., Armonk, NY, USA). Descriptive statistics for all data are reported as mean ± standard deviation, unless otherwise noted. Prior to any statistical analyses, data were assessed for normality using histogram analyses and Shapiro-Wilks tests. Outliers were removed if the reported value exceeded a *z* score ≥3.0. Within- (i.e. baseline vs week 2 vs week 4) and between-group (βA vs PL) differences were assessed using a repeated measures mixed model analysis of variance with an *a priori* alpha level <0.05. Due to the ordinal nature of the RPE scale, non-parametric related and non-related samples analyses of variance were used to determine differences within and between groups. The alpha level was adjusted based on the number of comparisons using a Bonferroni adjustment factor (i.e. 0.05 ÷ # of comparisons). To assess differences in VO_2peak_ within and between groups, a repeated measures analysis of co-variance was used with the change in individual weight, for each respective comparison, serving as a co-variate.

The practical magnitudes of difference for between- and within-groups were determined by Cohen’s *d* and *d*_*rm*_ effect size statistics [26] and categorized with Hopkin’s scale of magnitude for small sample sizes [27]. For RPE, effect sizes were determined using a *z* score transformation to Cohen’s *d* [26]. The scale of magnitude was as follows: trivial effect <0.20, small effect 0.20–0.59, moderate effect 0.60–1.19, large effect 1.20–1.99, and very large effect ≥2.0. Furthermore, smallest worthwhile change (SWC) was quantified to account for day-to-day variability within each outcome variable to further demonstrate clinical change values. SWC was calculated using the standard deviation multiplied by a small effect size for elite athletes of 0.20 for each outcome variable [28]. While *p* values have been included, the American Statistical Association does not recommend using *p* value cutoff points (i.e. <0.05) as the basis for determining meaningfulness or importance of an effect [29]. Lastly, to assist with data interpretation, percentage change, from baseline, for primary performance and physiological outcomes were calculated (i.e. percent change = ([post – baseline]/baseline) * 100).

## 3. Results

While 27 subjects participated in the study, only 23 (10 males, 13 females) successfully completed the entire four-week intervention. One athlete experienced an injury unrelated to the current methodology, two individuals became ill (also unrelated to the current study) and could not complete the trials, and one was excluded for failure to follow the supplementation protocol. Of note, eight of the eleven subjects experienced mild paresthesia within 2 weeks of βA consumption, with no other adverse side effects reported. Additionally, all subjects had not used any dietary supplements in the 3-months prior and had not taken βA supplementation for at least 1 year. Thus, the displayed results were analyzed based on 23 elite adolescent runners (age 17.36 ± 2.17 years, height: 173.76 ± 9.04 cm, weight: 63.40 ± 10.44 kg). All runners included in the current study trained at least five times a week with an annual total distance of 1980 ± 683 km over the past 3 years.

Regarding the data, normality assessments for all continuous variables did not indicate any violations of assumptions to be considered parametric nor were any outliers discovered; thus, only RPE (i.e. an ordinal measurement) was analyzed using non-parametric tests. Based on baseline testing, there were no differences between the groups regarding initial anthropometrics (i.e. body height, body weight), age, or performance variables (for both the GXT and submaximal trials), except final RER during the GXT (Table 1). The difference in the final RER during the GXT was deemed moderate (*d* = 0.61); therefore, the initial RER for each subject was used as a covariate when comparing baseline to week 4 measurements.

**Table 1.** Changes over 4 weeks in aerobic capacity performance variables within adolescent runners when ingesting beta-alanine (*n* = 12) or placebo (*n* = 11).

	Group	Baseline	Week 2	Week 4	Baseline vs Week 2	Baseline vs Week 4	Week 2 vs Week 4	Between Conditions	SWC
		Mean ± SD	Mean ± SD	Mean ± SD	Δ ± SD *d*, *p* value	Δ ± SD *d*, *p* value	Δ ± SD *d*, *p* value	*d, p* value	SD *0.2
Height	PL	172.2 ± 9.0	172.2 ± 9.0	172.2 ± 9.0	–	–	–	—	—
	BA	175.3 ± 9.6	175.3 ± 9.6	175.3 ± 9.6	–	–	–		–
Weight	PL	61.7 ± 7.6	62.0 ± 8.3	61.8 ± 8.3	0.26 ± 0.85 0.35,0.99	0.06 ± 1.03 0.11, 1.0	–0.2 ± 0.84 0.24, 1.0	0.11, 0.78	1.52
	BA	64.9 ± 12.9	64.8 ± 13.0	64.8 ± 12.7	–0.02 ± 0.83 0.12, 1.0	–0.05 ± 0.92 0.12, 1.0	–0.03 ± 0.82 –, 1.0		2.58
HR_max_ [bpm]	PL	191 ± 6	192 ± 5	191 ± 6	1 ± 3 0.32, 0.89	0 ± 3 –, 1.0	1 ± 4 0.24, 1.0	0.28, 0.67	1.9
	BA	188 ± 11	188 ± 9	189 ± 8	0 ± 9 –, 1.0	1 ± 4 0.24, 1.0	1 ± 9 0.11, 1.0		2.2
VO_2peak_ [ml.min^−1.^kg^−1^]	PL	56.7 ± 7.1	54.9 ± 7.0	53.0 ± 8.1	–1.8 ± 4.1 0.42, 0.55	−3.6 ± 4.7 0.75, 0.14	−1.9 ± 4.2 0.42, 0.09	1.03, 0.03	1.3
	BA	55.3 ± 7.7	56.4 ± 7.2	56.0 ± 6.9	−1.1 ± 4.2 0.25, 0.84	0.7 ± 3.6 0.18, 0.02	−0.4 ± 4.4 0.09, 0.86		1.5
TTE [s]	PL	363.0 ± 54.5	373.2 ± 50.2	368.1 ± 47.4	10.2 ± 34.8 0.28, 1.0	5.1 ± 52.1 0.09, 1.0	−5.1 ± 32.7 0.15, 1.0	0.46, 0.29	10.4
	BA	361.1 ± 60.6	373.8 ± 73.0	384.7 ± 59.3	12.8 ± 29.7 0.49, 0.55	23.6 ± 21.4 1.05, 0.01	10.8 ± 26.2 0.40, 0.59		11.6
[La] 3-min [mmol.L]	PL	10.1 ± 2.8	9.6 ± 2.2	9.8 ± 3.8	–0.5 ± 2.1 0.22, 1.0	–0.3 ± 4.2 0.07, 1.0	0.2 ± 3.3 0.07, 1.0	0.57, 0.20	0.5
	BA	10.4 ± 2.1	10.7 ± 2.1	10.6 ± 1.6	0.3 ± 2.1 0.13, 1.0	0.2 ± 2.1 0.09, 1.0	−0.1 ± 2.1 0.04, 1.0		0.4
V_E__final [L.min^−1^]	PL	119.7 ± 30.6	120.1 ± 27.9	120.5 ± 29.8	0.4 ± 6.8 0.05, 1.0	0.8 ± 9.5 0.08, 1.0	0.4 ± 6.2 0.06, 1.0	0.37, 0.41	5.8
	BA	123.4 ± 25.3	124.3 ± 23.9	127.6 ± 19.0	0.9 ± 7.3 0.11, 1.0	4.2 ± 9.1 0.44, 0.47	3.3 ± 6.4 0.50, 0.35		4.8
RER_Final_	PL	1.13 ± 0.10	1.10 ± 0.08	1.13 ± 0.04	–0.03 ± 0.14 0.21, 1.0	0.005 ± 0.10 –, 1.0	0.03 ± 0.06 0.47, 0.26	0.02, 0.96	0.02
	BA	1.18 ± 0.06	1.13 ± 0.11	1.18 ± 0.08	−0.05 ± 0.13 0.37, 0.69	0.003 ± 0.09 –, 1.0	0.05 ± 0.06 0.81, 0.05		0.01

Between condition effect sizes and *p* values compared the Δ ± SD of Baseline vs Week 4 between groups. VO_2peak_: peak oxygen uptake.

HR_peak_: peak heart rate (beats per minute); TTE: time to exhaustion; [La] 3-min: lactate concentration 3-minutes posttest; RER: respiratory exchange ratio (VCO_2_:VO_2_); V_E_: minute ventilation; VCO_2_: volume of expired carbon dioxide; PL: placebo group; BA: beta-alanine group; *d*: effect size; SWC: smallest worthwhile change (0.2 * baseline SD); ‘—‘: indicates an effect size of zero.

### 3.1. Maximal graded exercise testing

**Figure 2.**
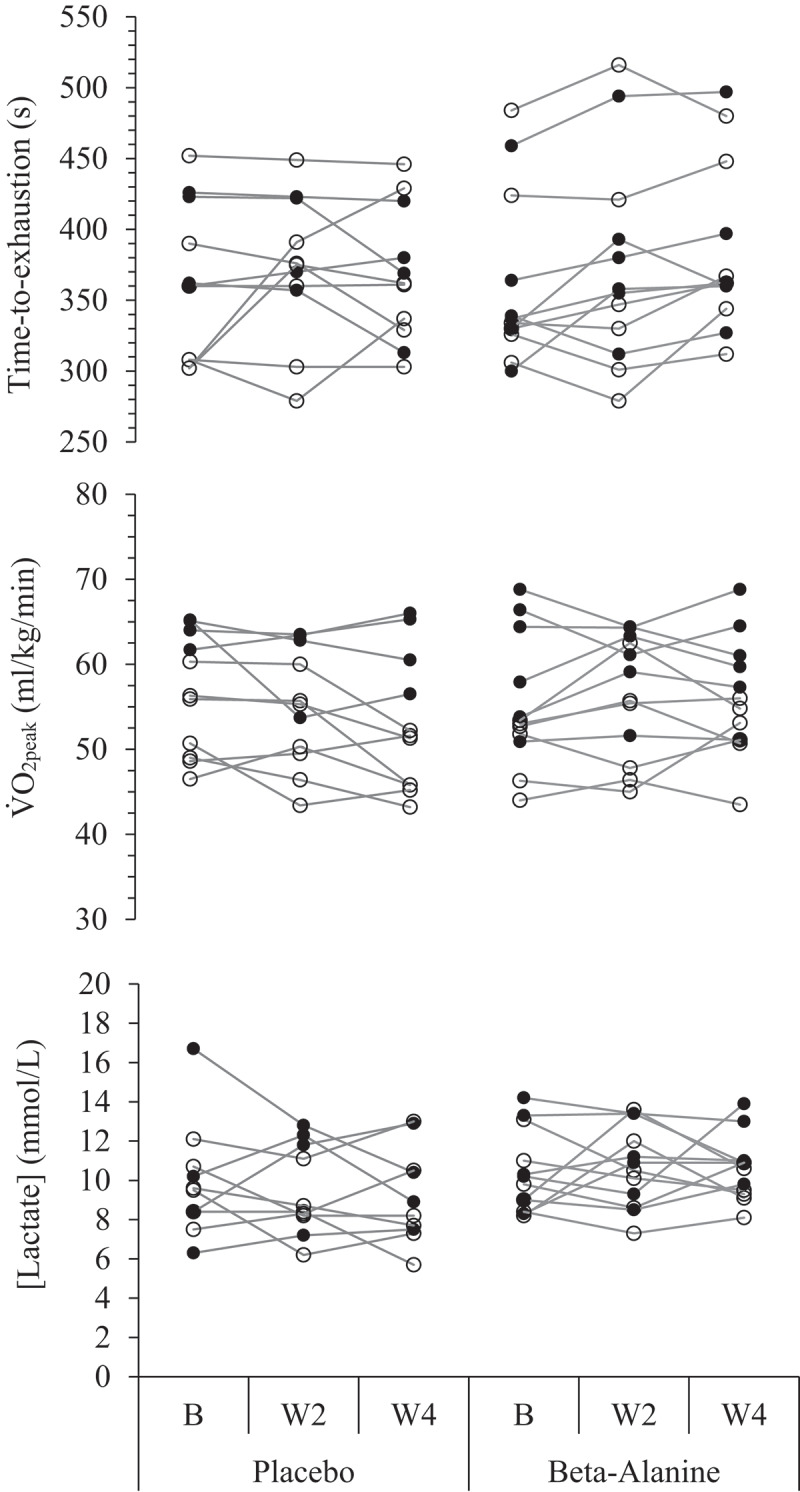
Individual changes in time-to-exhaustion, aerobic capacity (VO_2peak_), and lactate (3-min post) during a graded exercise test over a 4-week placebo (*n* = 11) or beta-alanine (*n* = 12) supplementation intervention in trained adolescent middle- and long-distance runners (aged 15–19 years).

Over the four-week intervention, there was a trivial difference in RER (*d* = 0.02), small effects in HR_max_ (*d* = 0.28), V_E_ (*d* = 0.37), [La] (*d* = 0.57), and TTE (*d* = 0.46), and a moderate effect in VO_2peak_ (*d* = 1.03) between groups favoring βA supplementation (Table 1). From pre- to post-intervention, the βA group experienced a trivial increase in VO_2peak_ by an average of 0.7 ± 3.6 ml.kg^−1.^min^−1^ (*d* = 0.18), while the PL group demonstrated a moderate decrement in VO_2peak_ performance of 3.6 ± 4.7 ml.kg^−1.^min^−1^ (*d* = 0.75). For the PL group, the individual weight changes from baseline to week 4 displayed a low strength, positive relationship with changes in VO_2peak_ (*r* = 0.48, *R*^*2*^ = 0.23), while the βA group showed a moderately high strength, negative relationship (*r* = −0.65, *R*^*2*^ = 0.42). Regarding time-to-exhaustion, there was a trivial increase of ~5 s within the PL group (+1.4%; *d* = 0.09); however, the βA group increased TTE by 23.6 ± 21.4 s (+6.5%, *d* = 1.05). Lastly, the results indicated an average increase of V_E_ by 4.2 ± 9.1 L.min^−1^ (*d* = 0.44) for the βA group during the GXT, with the PL group increasing V_E_ by an average of 0.8 ± 9.5 L.min^−1^ (*d* = 0.08). Figure 2 demonstrates the individual GXT changes in VO_2_, [La], and TTE in response to either the PL or βA supplementation over the course of 4 weeks.

Males are represented by filled-in circles, while females are open circles. B: baseline; W2: week 2; W4: week 4.

### 3.2. Ventilatory thresholds (VT1 & VT2)

**Figure 3.**
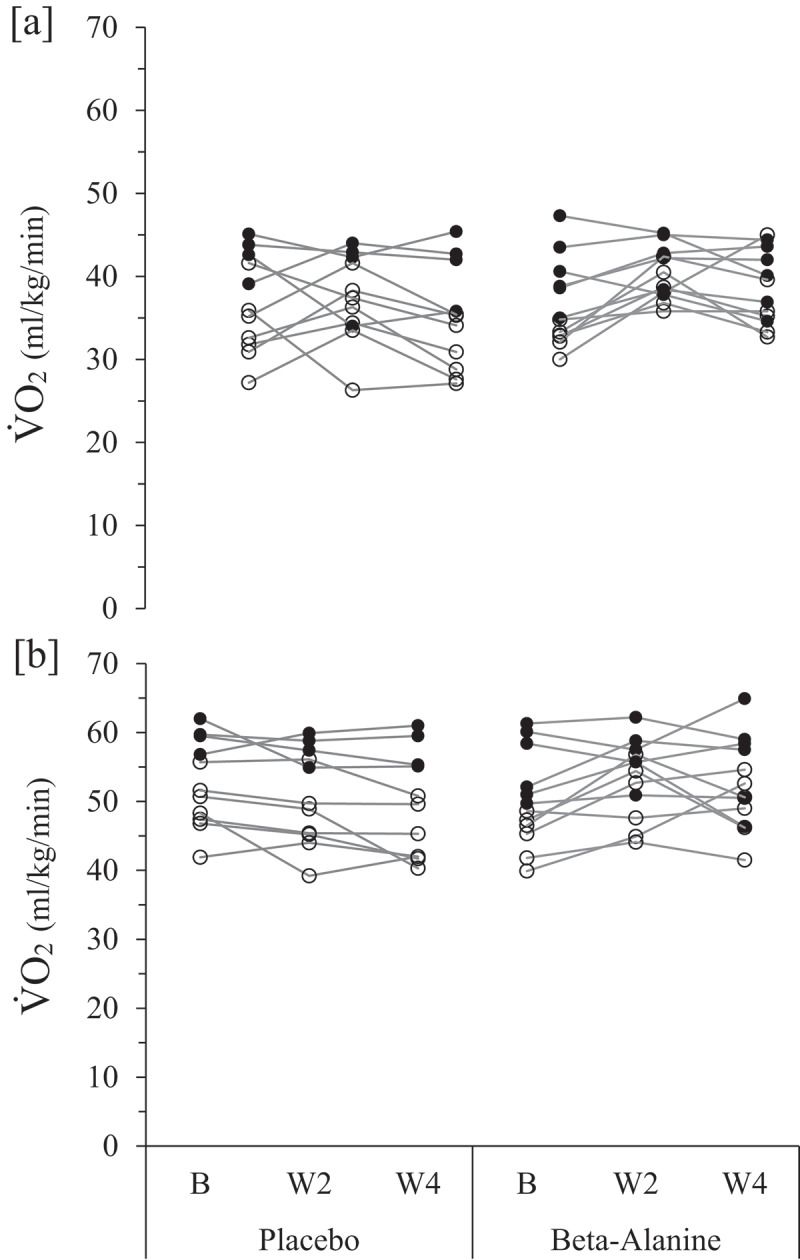
Individual changes in VO_2_ at the [A] first (VT1) and [B] second ventilatory threshold (VT2) during a 4-week placebo (*n* = 11) or beta-alanine (*n* = 12) supplementation intervention in trained adolescent middle- and long-distance runners (aged 15–19 years).

Table 2 displays the average resultant values for VO_2_, RER, velocity, and V_E_ at VT1 and VT2 for the PL and βA groups, while Figure 3 demonstrates individual changes. Regarding VT1, moderate practical differences were observed between the groups for VO_2_ (*d* = 0.99) and V_E_ (*d* = 0.71), while a small and trivial effect were shown for RER (*d* = 0.30) and velocity (*d* = 0.19), respectively. Additionally, small practical effects observed within the βA group for VO_2_ (+5%; *d* = 0.36), RER (+3.4%; *d* = 0.58), and velocity (+5.3%; *d* = 0.40), and a moderate effect for V_E_ (+12.1%; *d* = 0.78). For the PL group, small practical effects were shown for VO_2_ (−5%; *d* = 0.45), velocity (+3.3%; *d* = 0.32), and V_E_ (+3.4%; *d* = 0.39), along with a trivial decrease in RER (+1.1%, *d* = 0.14).

Males are represented by filled-in circles, while females are open circles. B: baseline; W2: week 2; W4: week 4.

**Table 2.** Changes in ventilatory thresholds over 4 weeks in adolescent runners for the placebo (*n* = 11) and beta-alanine (*n* = 12) groups.

	Group	Baseline	Week 2	Week 4	Baseline vs Week 2	Baseline vs Week 4	Week 2 vs Week 4	Between Conditions	SWC
***VT1***		Mean ± SD	Mean ± SD	Mean ± SD	Δ ± SD *d*, *p* value	Δ ± SD *d*, *p* value	Δ ± SD *d*, *p* value	*d, p* value	SD *0.2
VO_2_ [ml.min^−1.^kg^−1^]	PL	36.9 ± 5.7	37.4 ± 5.0	35.0 ± 6.0	0.5 ± 5.8 0.09, 1.00	−1.9 ± 4.2 0.45, 0.56	−2.4 ± 3.3 0.74, 0.14	0.99, 0.09	1.13
	BA	36.6 ± 5.0	40.3 ± 3.0	38.6 ± 4.2	3.7 ± 5.0 0.98, 0.02	2.0 ± 3.7 0.36, 0.80	−1.7 ± 3.5 0.50, 0.40		0.99
RER	PL	0.88 ± 0.06	0.85 ± 0.04	0.89 ± 0.03	−0.03 ± 0.08 0.37, 0.77	0.01 ± 0.07 0.14, 1.0	−0.14 ± 0.08 1.70, <0.001	0.30, 0.59	0.01
	BA	0.88 ± 0.05	0.86 ± 0.07	0.91 ± 0.04	−0.02 ± 0.08 0.23, 1.00	0.03 ± 0.05 0.58, 0.32	0.05 ± 0.06 0.77, 0.08		0.01
Velocity [km/hr]	PL	9.0 ± 1.4	9.4 ± 1.0	9.3 ± 1.3	0.4 ± 1.0 0.41, 0.64	0.3 ± 0.9 0.32, 0.95	−0.1 ± 0.7 0.13, 1.00	0.19, 0.71	0.28
	BA	9.5 ± 1.7	10.3 ± 1.2	10.0 ± 1.4	0.8 ± 0.8 1.00, 0.01	0.5 ± 1.2 0.40, 0.65	−0.3 ± 0.8 0.37, 0.67		0.33
[L]	PL	61.1 ± 15.0	63.7 ± 14.1	63.2 ± 16.3	2.6 ± 5.7 0.46, 0.53	2.1 ± 5.4 0.39, 0.72	−0.5 ± 4.5 0.11, 1.00	0.71, 0.13	3.00
	BA	62.2 ± 12.8	67.3 ± 12.3	69.7 ± 13.6	5.2 ± 7.2 0.71, 0.11	7.6 ± 9.6 0.78, 0.07	2.4 ± 7.6 0.31, 0.96		2.56
***VT2***									
VO_2_ [ml.min^−1.^kg^−1^]	PL	52.8 ± 6.1	50.9 ± 6.6	49.3 ± 7.2	−1.9 ± 3.4 0.56, 0.32	−3.4 ± 3.8 0.92, 0.05	−1.5 ± 3.1 0.51, 0.45	1.34, 0.006	1.22
	BA	50.2 ± 6.6	53.4 ± 5.4	52.6 ± 6.3	3.3 ± 4.1 0.78, 0.06	2.4 ± 4.8 0.50, 0.36	−0.85 ± 5.3 0.15, 1.00		1.31
RER	PL	1.06 ± 0.08	1.02 ± 0.06	1.07 ± 0.04	−0.04 ± 0.10 0.38, 0.79	0.01 ± 0.08 0.13, 1.0	0.05 ± 0.04 1.20, 0.01	—, 0.86	0.02
	BA	1.08 ± 0.05	1.05 ± 0.07	1.09 ± 0.06	−0.03 ± 0.09 0.35, 0.81	0.01 ± 0.07 0.14, 1.00	0.04 ± 0.08 0.50, 0.54		0.01
Velocity [km/hr]	PL	13.3 ± 1.7	13.2 ± 1.8	13.1 ± 1.7	−0.2 ± 0.8 0.12, 1.00	−0.2 ± 0.9 0.21, 1.00	−0.05 ± 0.7 0.15, 1.00	0.89, 0.05	0.33
	BA	13.2 ± 1.4	13.8 ± 1.5	13.8 ± 1.7	0.6 ± 0.6 0.96, 0.02	0.6 ± 0.9 0.63, 0.16	−0.03 ± 0.8 —, 1.00		0.29
V_E_ [L]	PL	101.6 ± 21.5	102.0 ± 23.2	102.5 ± 24.9	0.4 ± 8.2 0.05, 1.0	0.9 ± 7.4 0.12, 1.0	0.6 ± 5.6 0.09, 1.0	0.70, 0.13	4.29
	BA	101.4 ± 23.3	109.3 ± 21.6	109.7 ± 20.0	7.9 ± 6.3 1.25, 0.01	8.3 ± 13.1 0.64, 0.18	0.4 ± 12.3 0.03, 1.00		4.66

Between condition effect sizes and *p* values compare the Δ ± SD of condition. VT: ventilatory threshold; VO_2_: oxygen uptake. Baseline vs Week 4 RER: Respiratory Exchange Ratio (VCO_2_: VO_2_); VCO_2_: volume of expired carbon dioxide; V_E_: minute ventilation; PL: placebo group.

BA: beta-alanine group; SD: standard deviation; *d*: effect size; SWC: smallest worthwhile change (0.2*baseline SD); indicates an effect size of 0.

For VT2, a large practical change was demonstrated for VO_2_ (*d* = 1.34), along with moderate effects for velocity (*d* = 0.89) and V_E_ (*d* = 0.70) favoring the βA group, when compared to PL, over the four-week intervention (Table 2). The PL group experienced a 6.6% decrease in average VO_2_ at VT2 from baseline (*d* = 0.92), while the βA group increased VO_2_ at VT2 by 4.8% (*d* = 0.50). While the βA group displayed moderate practical effects for V_E_ (+8.2%; *d* = 0.63) and velocity (+4.5%; *d* = 0.64) at VT2, the PL group exhibited a trivial and small effect for V_E_ (+0.9%; *d* = 0.12) and velocity (−1.5%; *d* = 0.21), respectively. Furthermore, there were no between- or within-group changes for RER.

### 3.3. Submaximal testing (SMT)

**Figure 4.**
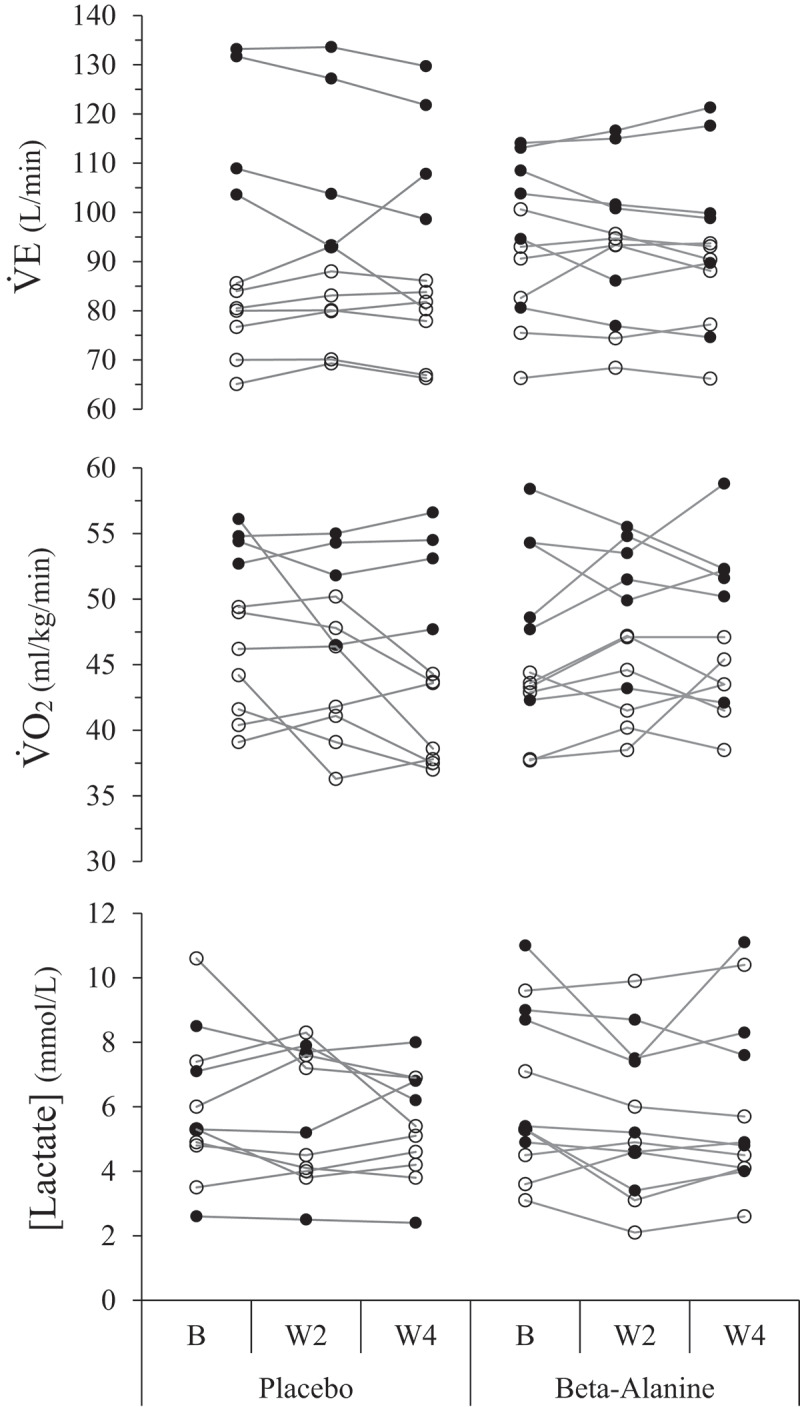
Individual changes in blood lactate concentration 3-minutes post 1500-meter run over a 4-week placebo (*n* = 11) or beta-alanine (*n* = 12) supplementation intervention in trained adolescent middle- and long-distance runners (aged 15–19 years).

Table 3 shows the mean (±SD) for the SMT results over the four-week intervention for the PL and βA groups. Over the intervention period, moderate practical differences in average HR (*d* = 0.73), VO_2_ (*d* = 1.07), and RER (*d* = 0.64) were observed between the groups. However, within-group changes for PL demonstrated trivial differences for V_E_ (*d* = 0.11), and RPE (*d* = 0.13). When examining average VO_2_ during the SMT, the PL group showed an average decrease of −3.0 ± 3.9 ml.kg^−1.^min^−1^ (*d* = 0.77), despite a small mean increase for the βA group (0.9 ± 3.4 ml.kg^−1.^min^−1^; *d* = 0.25). Individual weight changes from baseline to week 4 displayed a low strength, positive relationship with changes in VO_2_ for the PL group (*r* = 0.42, *R*^*2*^ = 0.18), while the βA group displayed a moderately strength, negative relationship (*r*=-0.58, *R*^*2*^ = 0.34). Figure 4 displays the individual changes in the 1500-m run performance variables (i.e. HR, VO_2_, and [La]) for the PL and βA group over the four-week intervention.

Males are represented by filled-in circles, while females are open circles. $\dot{\;}V$E: minute ventilation; $\dot{\;}V$O_2peak_: average oxygen consumption; B: baseline; W2: week 2; W4: week 4.

**Table 3.** Changes in performance variables during a 1500-m submaximal treadmill run in adolescent runners (*n* = 23).

	Group	Baseline	Week 2	Week 4	Baseline vs Week 2	Baseline vs Week 4	Week 2 vs Week 4	Between Conditions	SWC
		Mean ± SD	Mean ± SD	Mean ± SD	Δ ± SD *d*, *p* value	Δ ± SD *d*, *p* value	Δ ± SD *d*, *p* value	*d, p* value	SD *0.2
HR_avg_ [bpm]	PL	176 ± 4	175 ± 7	176 ± 5	−1 ± 6 0.16, 1.00	0 ± 3 —, 1.00	1 ± 6 0.15, 1.00	0.73, 0.10	1
	BA	174 ± 12	170 ± 10	167 ± 10	−4 ± 6 0.63, 0.10	−7 ± 13 0.48, 0.26	−3 ± 11 0.26, 1.00		3
VO_2__avg [ml.min^−1.^kg^−1^]	PL	48.0 ± 6.1	46.4 ± 6.2	44.9 ± 7.2	−1.6 ± 3.7 0.41, 0.60	−3.0 ± 3.9 0.77, 0.09	−1.4 ± 3.3 0.44, 0.58	1.07, 0.02	1.2
	BA	46.3 ± 6.6	47.3 ± 5.8	47.2 ± 5.9	1.0 ± 3.1 0.31, 0.90	0.9 ± 3.4 0.25, 1.00	−0.1 ± 3.3 0.03, 1.00		1.3
[La] 3-min [mmol.L]	PL	6.0 ± 2.3	5.7 ± 2.1	5.5 ± 1.7	−0.3 ± 1.3 0.22, 1.00	−0.5 ± 1.4 0.33, 0.84	−0.2 ± 1.2 0.16, 1.00	—, 0.91	0.5
	BA	6.5 ± 2.6	5.6 ± 2.4	6.0 ± 2.7	−0.8 ± 1.2 0.70, 0.13	−0.5 ± 1.3 0.38, 0.79	0.4 ± 1.2 0.33, 0.87		0.5
$\dot{V}$_E__avg [L.min^−1^]	PL	92.7 ± 23.5	92.8 ± 21.2	91.0 ± 21.0	0.2 ± 4.9 0.02, 1.00	−1.7 ± 5.1 0.33, 0.99	−1.8 ± 6.4 0.27, 1.00	0.11, 0.80	4.7
	BA	93.6 ± 15.2	93.1 ± 14.9	92.5 ± 16.0	−0.5 ± 5.1 0.10, 1.00	−1.1 ± 5.6 0.19, 1.00	−0.5 ± 3.2 0.18, 1.00		3.0
RER_avg_	PL	1.03 ± 0.11	1.01 ± 0.06	1.04 ± 0.04	−0.02 ± 0.11 0.18, 1.00	0.01 ± 0.10 0.10, 1.00	0.04 ± 0.06 0.46, 0.21	0.64, 0.17	0.02
	BA	1.07 ± 0.07	0.99 ± 0.05	1.03 ± 0.03	−0.08 ± 0.07 0.98, 0.02	−0.04 ± 0.05 0.69, 0.21	0.04 ± 0.05 0.76, 0.01		0.01
RPE_avg*	PL	12(2)	13(5)	13(6)	1(3) 0.78, 0.23	1(2) 0.10, 0.86	0(2) 0.92, 0.17	0.13, 0.12	1
	BA	12(2)	12(3)	12(4)	0(3) 0.59, 0.33	0(2) 1.59, 0.03	0(2) 0.38, 0.52		1

*RPE_avg: average rating of perceived exertion (reported as median (interquartile range)). —: indicates an effect size of zero.

Between conditions: effect sizes and *p* values compared the Δ ± SD of Baseline vs Week 4 condition between the P and BA groups.

PL: placebo group (*n* = 11); BA: beta-alanine group (*n* = 12); VO_2__avg: average oxygen uptake; HR_avg_: average heart rate (beats per minute).

[La] 3-min: lactate concentration 3-minutes posttest; RER: respiratory exchange ratio (VCO_2_: VO_2_); V_E__avg: average minute ventilation.

VCO_2_: volume of expired carbon dioxide; VO_2_: volume of inspired oxygen; *d*: effect size; SWC: smallest worthwhile change (0.2 * baseline SD).

## 4. Discussion

Previous research into βA supplementation has primarily examined adults, with information lacking on adolescent athletes. Therefore, the purpose of this investigation was to determine the effect of four-weeks of βA consumption on submaximal and maximal performance in elite adolescent runners. The main findings indicated that despite a trivial increase in VO_2peak_, four-weeks of βA supplementation increased TTE during maximal-intensity exercise by 6.5% compared to a 1.4% increase in the PL group. Additionally, there was a large practical effect (*d* = 1.34) regarding the VO_2_ at which VT2 occured between groups, increasing by an average of 2.4 ± 4.8 ml.kg^−1.^min^−1^ for the βA group compared to an average decrease of −3.4 ± 3.8 ml.kg^−1.^min^−1^ in the PL group. Regarding the submaximal running performance (i.e. timed 1500-m run), βA reduced average HR by 7 bpm (*d* = 0.48) and RER by 0.04 (*d* = 0.69) compared to no change in the PL group. During the submaximal running, both groups demonstrated trivial-to-small adaptations for [La] and V_E_. These findings suggest that βA supplementation may be a useful ergogenic strategy for endurance coaches working with adolescent athletes. While it may not increase maximal aerobic capacity, it appears to enhance performance by prolonging TTE and improving tolerance to higher-intensity segments during competition.

The current findings agree with previous research indicating that βA supplementation increases TTE, despite trivial-to-no effect on VO_2peak_ during aerobic performance [12–17,30]. For instance, after 6 weeks of high-intensity interval training, recreationally trained college-aged males, consuming 6 g.day^−1^ βA daily, experienced an 18.7% increase in TTE during a VO_2peak_ test, compared to a 15.1% increase in the PL group [12]. However, changes in VO_2peak_ were similar when comparing βA (i.e. 11.9%) to PL (i.e. 12.6%). Similarly, 38 recreationally active males and females saw a 2.5% decrement in VO_2peak_ (i.e. −1 ml.kg^−1.^min^−1^) after 4 weeks of βA supplementation, at ~6 g.day^−1^, despite a 3.2% increase in TTE [17]. Lastly, ~5.6 g.day^−1^ of βA supplementation for 4 weeks did not affect cycling VO_2peak_ (pre 1.91 ± 0.16 L.min^−1^ versus post 1.91 ± 0.15 L.min^−1^) in adult females [14]. Yet, TTE improved by an average of 2.6% (i.e. 29.18 s) compared to no change in the PL group (i.e. 18 min 53.6 s pre versus 18 min 53.4 s post). It is important to note that while individuals within the current study increased VO_2peak_ by an average of 1.6%, the observed day-to-day variation of VO_2peak_ testing is ~2.6% [31]. Thus, it cannot be stated that 4 weeks of βA supplementation had an effect on aerobic capacity.

In addition to increases in TTE, the βA group experienced a delayed onset of VT2 via increases in VO_2_ and velocity by 4.78% and 4.54%, respectively, compared to the PL group which experienced a 6.6% decrease in VO_2_ (*d* = 1.34) and 1.5% decrease in velocity (*d* = 0.89). For VT1, within-subjects changes for VO_2_, RER, velocity, and V_E_ were determined to be small, apart from V_E_ for the BA group being a moderate effect (*d* = 0.78). These results support previous work demonstrating increases in VO_2_ (i.e. 7%) and power out (i.e. 10%) at VT2 for males supplementing with βA for 4 weeks [30]. Whereas the PL group saw a 6% decrease in VO_2_ and 2% decrease in power out at VT2, despite a 4.6% increase in VO_2peak_. Similarly, adult females supplementing with βA for 4 weeks exhibited a 16% increase in VO_2_ (in L.min^−1^) and 14% greater watts at VT2 during a cycling GXT [14].

Regarding the SMT (i.e. 1500-m run), results of the current investigation indicated moderate differences in average HR (*d* = 0.73), VO_2_ (*d* = 1.07), and RER (*d* = 0.64) between groups. While only trivial differences were observed between groups for RPE (*d* = 0.13) during the SMT, these results are consistent with a previous meta-analysis demonstrating trivial-to-no effects of βA on RPE [32]. Although, when examining within-group changes over the four-week period, βA supplementation did not appear to influence average VO_2_ as the βA group experienced a 1.94% increase (0.4 ml.kg-1.min-1 below the smallest worthwhile change). However, the PL group decreased average VO_2_ during the SMT by 6.45% despite a trivial change in V_E_ between groups (*d* = 0.11). While VO_2_ may have not been affected, the βA group decreased average HR by 4% during the SMT with no change for the PL group. These results are in slight agreement with previous literature examining the effect of 28 days of βA supplementation on work output at various heart rate threshold points during cycle ergometry [17]. Previous findings showed that the βA and PL groups experienced similar decrements in HR (i.e. 3 bpm and 2 bpm, respectively) for a given submaximal workload (i.e. 60% maximal workload); however, the βA group increased work completed (in Watts) by 5.6%, versus a 5.7% decrease in the PL group [17].

These collective results indicate that βA supplementation may provide less benefits to activities performed at lower intensities (e.g. VT1), compared to higher intensities (e.g. VT2, VO_2peak_ workloads) where H^+^ buffering capacity is of greater concern. For example, in a study of 22 water polo athletes (aged 17–20 years), there were trivial-to-no changes in repeated sprint ability (i.e. 6 × 10 m sprints) after 28 days of βA consumption [22]. While unmeasured, the improvements in TTE, RER, and HR may be explained by potential increased levels of skeletal muscle carnosine. As βA is the rate-limiting precursor in carnosine synthesis, long-term use of βA supplementation (i.e. >4 weeks) has been shown to increase carnosine levels by ~64%, and continues to rise to ~80% after 10 weeks of use [10,11]. Carnosine functions as a buffer for H^+^ and reactive oxygen species during exercise; thereby attenuating reductions in pH during high-intensity activities, resulting in improved muscle function and increased TTE [2,5,6,8,14].

Additionally, increased muscle H^+^ buffering may potentially increase glycolytic energy production during the final stages of graded exercise (or high intensity) performance equaling higher peak [La] in active muscle tissue and blood [33]. For instance, in the present study, post-GXT [La] increased in the βA group by 1.92% (i.e. 0.2 mmol.L^−1^) and decreased by 2.98% (i.e. 0.2 mmol.L^−1^) in the PL group, showing a moderate effect (*d* = 0.57) between groups. However, [La] 3-min after the 1500-m run demonstrated that the βA group experienced a 7.7% decrease in [La] compared to 8.3% in the PL group. Despite these adaptations, the alterations in [La] post exercise, for both the GXT and 1500-m run, did not exceed the smallest worthwhile change scores indicating that the fluctuations in [La] may have been caused by day-to-day variance, and not βA supplementation. These findings are consistent with a previous meta-analysis examining [La] after various athletic events (e.g. cycling, running, and rowing) [32]. Of the 11 studies included within the meta-analysis, athletes supplementing with βA demonstrated trivial changes in [La] post-activity [32].

While βA supplementation may attenuate the rise in H^+^ ions, the accumulation of muscle and blood [La] and production of H^+^ are the result of an independent mechanism [34]. Despite previous notions, H^+^ ions are released within glycolysis as a result of the conversion of fructose-6-phosphate to fructose-1,6-bisphate, as well as the conversion of glyceradehyde-3-phosphate to 1,3-bisphosphoglycerate, and not during La production. The production of La (through the buffering of H^+^ via pyruvate) consumes and does not release, H^+^ ions; thus, the trivial effect of βA supplementation on [La] was expected.

While the results of the current study showed that βA supplementation increased TTE, with no subsequent effect on VO_2peak_, this investigation is not without limitations. For instance, subjects only consumed the βA supplementation for a period of 4 weeks. However, research indicates that 4 weeks is the minimum threshold to experience beneficial adaptations as a result of βA intake [8]; thus, longer intervention periods may be required to experience improvements in VO_2peak_ and other performance variables. Second, skeletal muscle carnosine levels were not directly measured, although it has been previously reported that four-weeks of βA supplementation may increase carnosine levels by up to ~64% [2,6,10]. Another limitation is body weight fluctuations occurring over the course of the study, accompanied with day-to-day variations of VO_2peak_. For example, the individual weight changes in the PL group, from baseline to week 4, displayed a low strength, positive relationship with changes in VO_2peak_ (*r* = 0.48, *R*^*2*^ = 0.23), while the βA group showed a moderately high strength, negative relationship (*r*=-0.65, *R*^*2*^ = 0.42), predicting 42% of the change in VO_2peak_. Therefore, the individual changes in VO_2peak_ for the βA group may not be fully attributable to the supplementation.

## 5. Practical applications

The present study was designed to determine the effect of 28 days of βA supplementation on submaximal and maximal endurance performance in elite adolescent runners. The current results agree with previous research examining adult athletes such that long-term ingestion of βA may prolong TTE and increase submaximal ventilatory thresholds. Based on these findings, practitioners should note that βA may potentially assist in aerobic endurance performance for elite adolescent runners. Although, improvements in VO_2peak_ may not be observed, the potential mechanism for prolonging TTE is likely an increase in intracellular H^+^ buffering via a rise in muscle carnosine levels. While increased buffering capacity is a potential mechanism, a limitation of this study is that muscle carnosine levels were not measured. Thus, future research should examine the direct effect of βA supplementation on carnosine accumulation in adolescents as increases may differ based on maturation and training status. Additionally, the current study only examined 4 weeks of supplementation, whereas future research should examine longer durations of use (e.g. ≥8 weeks) in adolescent athletes to determine changes in aerobic performance.
